# Publisher Correction: Diverse populations of local interneurons integrate into the Drosophila adult olfactory circuit

**DOI:** 10.1038/s41467-018-07001-7

**Published:** 2018-11-06

**Authors:** Nan-Fu Liou, Shih-Han Lin, Ying-Jun Chen, Kuo-Ting Tsai, Chi-Jen Yang, Tzi-Yang Lin, Ting-Han Wu, Hsin-Ju Lin, Yuh-Tarng Chen, Daryl M. Gohl, Marion Silies, Ya-Hui Chou

**Affiliations:** 1 Institute of Cellular and Organismic Biology, Academia Sinica, Taipei 11529, Taiwan; 2 Department of Neurobiology, Stanford University, Stanford, CA 94305 USA; 3 Neuroscience Program of Academia Sinica, Academia Sinica, Taipei 11529, Taiwan; 4 Present Address: Research Institute of Molecular Pathology (IMP), Vienna Biocenter, Campus-Vienna-Biocenter 1, 1030 Vienna, Austria; 5 Present Address: University of Minnesota Genomics Center, 1–210 CCRB, 2231 6th Street SE, Minneapolis, MN 55455 USA; 6 Present Address: European Neuroscience Institute, University Medical Center Göttingen, Grisebachstr. 5, 37077 Göttingen, Germany

Correction to: *Nature Communications* **9**, 2232; 10.1038/s41467-018-04675-x; published online 8 June 2018

The original version of this Article contained errors in Figs. 4 and 6. In Fig. 4, panel a, text labels UAS-FLP and LexAop2>stop>myr::smGdP-HA were shifted upwards during typesetting of the figure, and in Fig. 6, panel h, the number 15 was incorrectly placed on the heat map scale. These have now been corrected in both the PDF and HTML versions of the Article.

