# Supplementary material for: Diverse populations of local interneurons integrate into the Drosophila adult olfactory circuit
Source: Nat Commun. 2018 Jun 8;9:2232. doi: 10.1038/s41467-018-04675-x (PMC5993751; doi:10.1038/s41467-018-04675-x)
Supplement: Supplementary file 1 — Supplementary Information [file 41467_2018_4675_MOESM1_ESM.pdf]

Supplementary Information

Diverse populations of local interneurons integrate into the *Drosophila* adult olfactory circuit

Liou et al.

Supplementary Note 1. The soma position of larval LNs.

Larval LNs form two clusters in the larval AL, one in the lateral region and the other in the ventral region (Fig. 2a, Supplementary Fig. 1a). Our data show that all *189Y*-positive larval LNs (in the ventral cluster) and *NP3056*-positive larval LNs (in both lateral and ventral clusters) reintegrate into the adult circuit. Furthermore, the reintegrated cells all have somas that are located in the lateral LN cluster of adult brains (Fig. 4c, e), suggesting that the positions of LN clusters in the larval AL and the adult AL are not directly related. Therefore, it is not clear whether the two larval LN clusters arise from the same or different lineages. In addition, *670*-positive larval LNs, which eventually degenerate, are found in the ventral cluster among *189Y*-positive and *NP3056*-positive larval LNs. Thus, the position of larval LN somas did not dictate whether the cells are pruned or degenerate.

Supplementary Note 2. The gap between the birth time of LNs and their emergence and integration to the olfactory circuit.

Previous studies have used clonal analyses to demonstrate that LNs are born from embryonic to late pupal stages[1-3]. Therefore the previous studies provided only the birth time of distinct types of LNs and no information about how and when those LNs develop and integrate to the circuit. In this work, we found a certain time delay between the birth time of a given LN and the emergence and integration of the LN to the circuit. In addition, not counting those LNs born at the embryonic stage, at least 48 lateral LNs are born during the larval stage and remain in the adult brains[2]. However we found that approximately 22 larval LNs survive metamorphosis and remain in the AL of 24 h APF pupae. In other words, it is likely that more than 26 adult-specific LNs (after deducting 22 LNs from 48 LNs born at the larval stage plus embryonic born LNs) in the lateral cluster emerge and integrate to the circuit after 24 h APF.

Supplementary Note 3. The types of larval LNs.

Four types of *189Y*-positive larval LNs in late 3rd instar larval brains were identified in this work (Fig. $4b_1$-b_4). They are either found with dense processes restricted to and covering the whole AL (Fig. $4b_1$, n = 22/68 single cell clones (SCCs)), covering the whole AL and innervating SEZ (Fig. $4b_2$, n = 30/68 SCCs), sparse processes covering the whole AL (Fig. $4b_3$, n = 9/68 SCCs), or dense processes covering a part of the AL (Fig. $4b_4$, n = 7/68 SCCs). The first and second types of *189Y*-positive larval LNs were reported previously[4]. Four types of *NP3056*-larval LNs in late 3rd instar larval brains were also identified in this work (Fig. $4d_1$-d_4). They either exhibit processes restricted to and covering the entire AL (Fig. $4d_1$,

dashed circle, n = 21/86 SCCs), covering a part of the AL and innervating the SEZ (Fig. 4d$_2$, contoured by white dashed line, n = 24/86 SCCs), covering the entire AL and innervating the SEZ (Fig. 4d$_3$, n = 25/86 SCCs), or with most processes in the AL and a few processes to dorsal brain regions (arrows) and the SEZ (Fig. 4d$_4$, n = 16/86 SCCs). Two types of *NP3056*-positive larval LNs shown in Fig. 4d$_2$ and Fig. 4d$_3$ were reported previously[4].

Supplementary Note 4. Glomerular index.

The glomerular index shown in figures are as follows:

(Fig. 6h, 6i, Supplementary Fig. 12a, 12b, 12c)

DA1, DA2, DA3, DA4m, DA4l, DM1, DM2, DM3, DM4, DM5, DM6, VA1d, VA1v, VA2, VA3, VA4, VA5, VA6, VA7m, VA7l, VM1, VM2, VM3, VM4, VM5v, VM5d, VM6, VM7, 1, D, DC1, DC2, DC3, DC4, DL1, DL2d, DL2v, DL3, DL4, DL5, V, VL1, VL2a, VL2p, VC1, VC2, VC3, VC4, DP1m, DP1l, VP1, VP2, VP3, Arm, Column

(Fig. 6j, Supplementary Fig. 8c right, 8e, 12d)

V, VM6, VA3, VM4, VM1, VL1, VA4, VA2, VA5, VM3, VA7m, VA7l, VP1, VM2, VP2, VC2, VC3, VA1v, VC4, VP3, VC1, VL2p, VM5v, VL2a, VA6, 1, DM5, VM7, VM5d, VA1d, Column, DC3, DM4, Arm, DA4l, DA2, DC2, DM2, DM6, DC4, DA4m, DL2v, DP1l, DC1, DA1, DL2d, DM1, DA3, DP1m, DM3, DL4, D, DL1, DL5, DL3

(Fig. 6k, Supplementary Fig. 8d right)

V, VA3, VL1, VA5, VA4, VM6, VM4, VA7l, VA2, VA7m, VA1v, VM1, VM3, VC2, VP3, VC4, VL2p, VC3, VA6, VM2, VL2a, VP1, VM5v, VC1, VA1d, VP2, DC3, 1, Column, VM5d, DA4l, DM5, VM7, DA2, DA4m, DA1, DM4, DL2d, Arm, DL2v, DC4, DC1, DC2, DA3, DM6, DP1l, DM2, D, DL4, DM1, DP1m, DL1, DL3, DM3, DL5

(Supplementary Fig. 8c left and middle, 8d left and middle, 8g left, 9d left and middle)

V, VM6, VA3, VL1, VM4, VA5, VA4, VA2, VM1, VM3, VA7m, VA7l, VA1v, VP1, VM2, VC2, VC3, VP3, VP2, VC4, VL2p, VL2a, VC1, VM5v, VA1d, 1, VA6, DM5, Column, VM7, DC3, DM4, VM5d, Arm, DA4l, DM6, DA2, DP1l, DC2, DA1, DL2d, DC1, DC4, DM2, DA4m, DL2v, DA3, DL4, DP1m, DM1, DM3, D, DL1, DL5, DL3

(Supplementary Fig. 9d right)

V, VM6, VL1, VM4, VA3, VM1, VA4, VA5, VA2, VM3, VP1, VP3, VA1v, VP2, VA7m, VA7l, VL2p, VC2, VC3, VM2, VC4, VC1, VL2a, VM5v, VA6, Column, VA1d, 1, DM5, VM7, Arm, DC3, VM5d, DM4, DL2d, DL2v, DA2, DA4l, DA1, DP1l, DC4, DC2, DM6, DM2, DC1, DA4m, DP1m, DM1, DA3, DL4, DM3, D, DL1, DL3, DL5

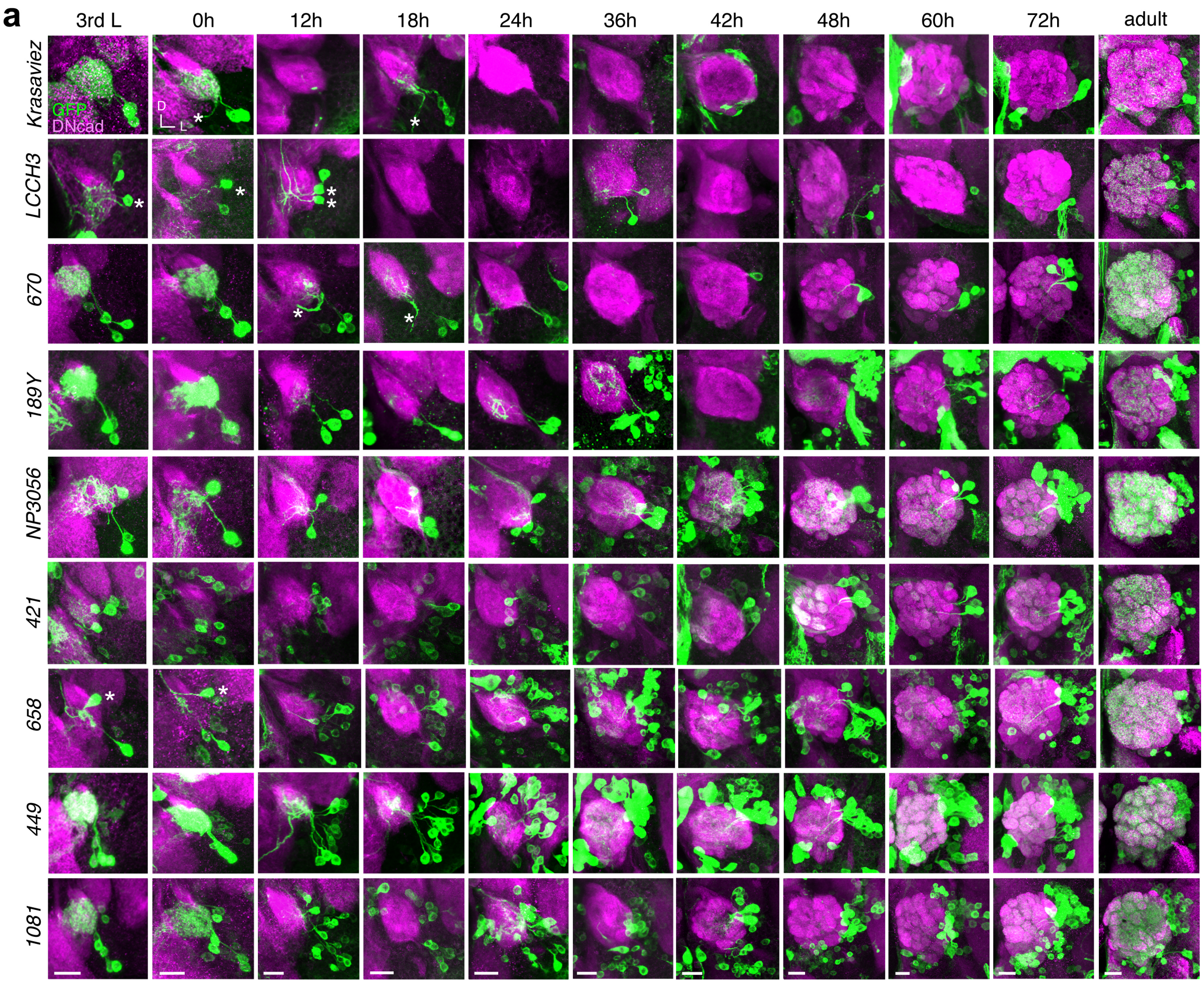
a
3rd L
0h
12h
18h
24h
36h
42h
48h
60h
72h
adult
Krasaviez
LCCH3
670
189Y
NP3056
421
658
449
1081
GFP
DNcad
D
L

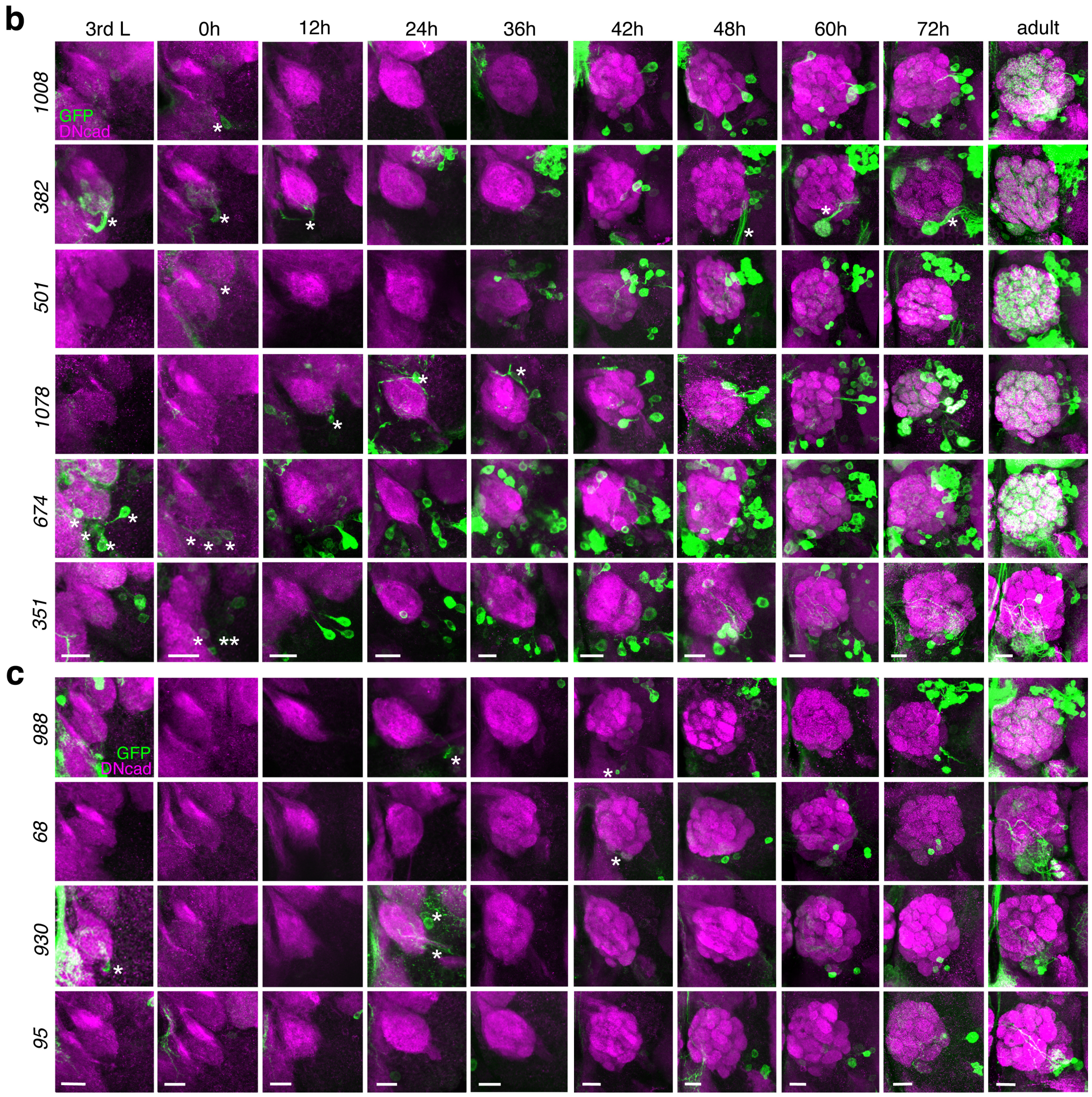

Supplementary Fig. 1 The development of distinct subsets of LNs. Projected confocal images show larval, pupal and adult brains stained with neuropil markers DNcad (magenta, larval and pupal brains) or Bruchpilot (magenta, adult brains). LNs were visualized by GAL4-driven mCD8GFP. Developmental stages are indicated by 3rd L: third larval instar, hours after puparium formation (APF): pupal stages, and adult. **a** Group 1: GAL4 lines that label LNs as early as late 3rd instar larvae. Identical images of *189Y*, *NP3056*, *449* and *670* are also shown in Fig. 2a. **b** Group 2: GAL4 lines labeling LNs that emerge from early to mid-pupal stages (48 h APF). Identical images of *351* and *501* are also shown in Fig. 2b. **c** Group 3: GAL4 lines labeling LNs that emerge after 48 h APF. Identical images of *95* and *988* are also shown in Fig. 2c. Asterisks denote glia or neurons that are not LNs. Scale bars, 20 μm. All ALs in this and following figures are oriented such that the midline is in the left. D, dorsal; L, lateral.

a

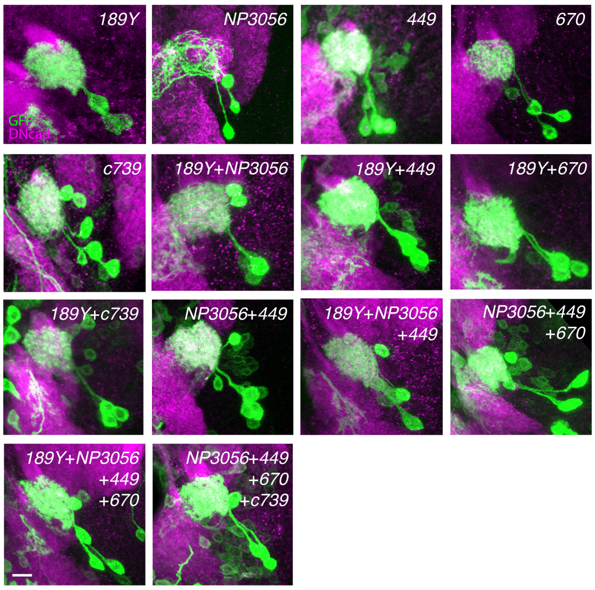

b

GAL4 lines	No of LNs	No of ALs
Single GAL4		
189Y	4.85 ± 0.67	26
NP3056	3.25 ± 0.71	8
449	15.90 ± 1.29	10
670	2.90 ± 0.32	10
c739	13.60 ± 2.95	10
Two GAL4s		
189Y + NP3056	6.25 ± 1.22	12
189Y + 449	18.73 ± 0.79	11
189Y + 670	4.19 ± 0.83 *	16
189Y + c739	12.0 ± 2.83	16
NP3056 + 449	19.14 ± 1.86	7
Three GAL4s		
189Y + NP3056 + 449	6.29 ± 1.07 *	14
NP3056 + 449 + 670	22.13 ± 1.13	8
Four GAL4s		
189Y + NP3056 + 449 + 670	5.56 ± 0.63 *	16
NP3056 + 449 + 670 + c739	15.93 ± 3.05 *	14

Supplementary Fig. 2 Estimated number of LNs labeled in individual and combined GAL4 lines. **a** Projected confocal images show GAL4-labeled LNs (GFP, green) and neuropil (DNcad, magenta). Scale bars, 20 μm. **b** Estimated number of labeled LNs per AL are shown as mean ± s.d., based on the scoring of 7-26 ALs. Asterisks denote strong genetic interaction among *GAL4* insertions in combined *GAL4* lines. From the results of two combined GAL4 drivers, *449* and *NP3056* label non-overlapping subsets of larval LNs. *189Y*-labeled larval LNs are either non-overlapping, or overlap by one LN with those labeled by *449*. Since *189Y*- and *449*-positive larval LNs innervate distinct regions of the AL at 24 h APF (Fig. 2a), these two GAL4s probably label non-overlapping subsets of larval LNs. Therefore, the estimated lower bound number of total larval LNs is either 26 or 27 (22 derived from *NP3056*+*449*+*670*-positive, plus five derived from *189Y*-positive, with or without one LN labeled by both *189Y* and one of the other GAL4 drivers). Note that *c739*-GAL4 labels a subset of larval LNs[5] that has strong genetic interaction with *189Y* and the combination of *NP3056*, *449* and *670*, thus precludes further estimation of larval LN number.

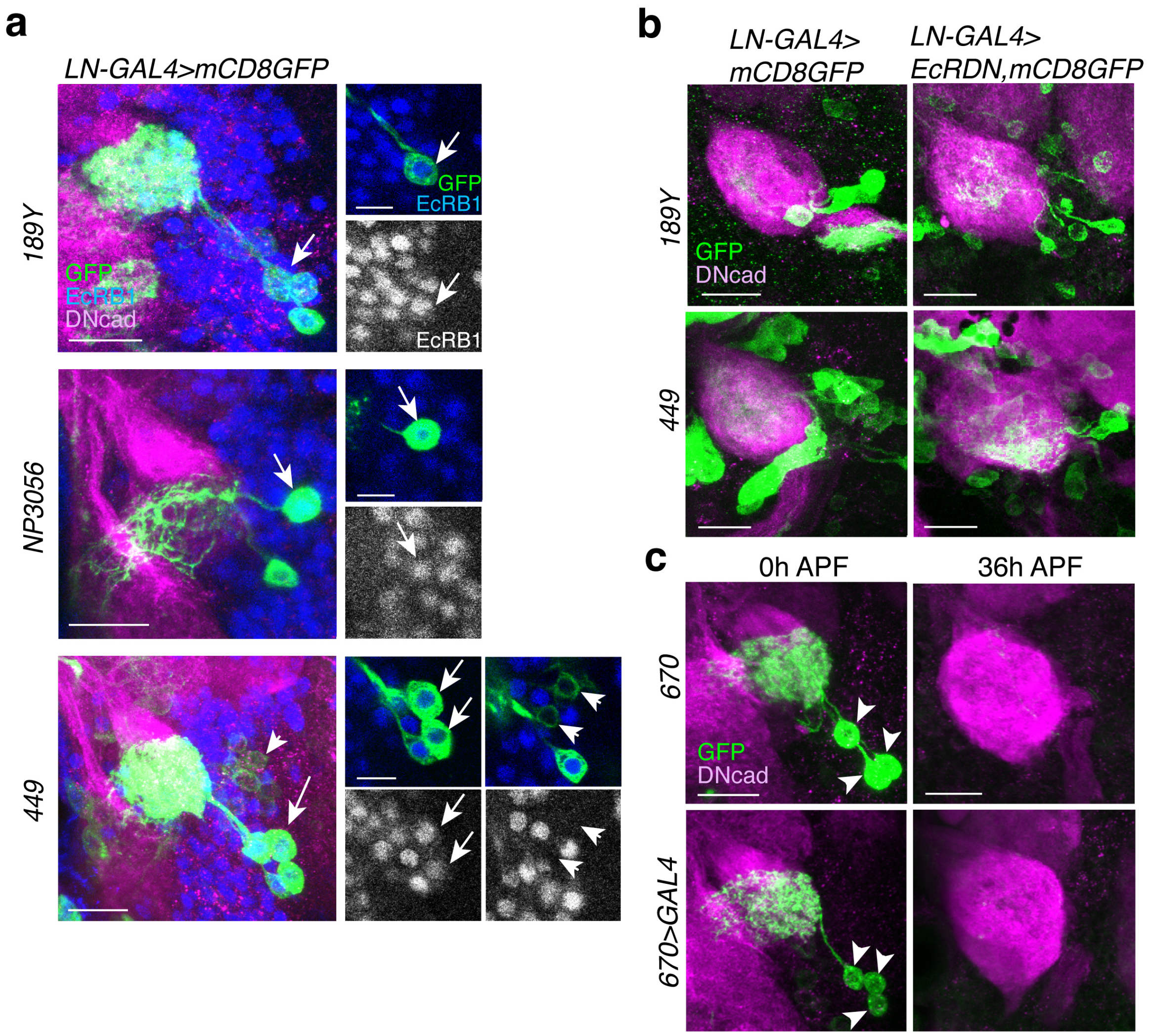

Supplementary Fig. 3 *189Y*-positive, *NP3056*-positive and *449*-positive larval LNs express EcRB1 and undergo pruning through the ecdysone signaling pathway. **a** (Left) Projected confocal image showing 0 h APF pupal brain carrying *189Y*-postive, *NP3056*-positive or *449*-positive larval LNs, co-stained with EcRB1 (blue) and DNcad (magenta). (Right) Single confocal section shows larval LNs (green) express EcRB1 (arrows, blue in the top panel and white in the bottom panel). A subset of *449*-positive larval LNs does not express EcRB1 (arrowheads). *n* = 11 brains for each *GAL4*. Scale bars, 20 μm (left panels) and 10 μm (right panels). **b** Blocking ecdysone signal in *189Y*-positive or *449*-positive larval LNs by overexpressing truncated Ecdysone receptor (EcR) leads to failure of pruning. Compared to *189Y*-LN processes, which occupy the medial region, *449*-LN processes occupy the central region of the AL in 24 h APF control pupal brains (*n* = 19 and 22 brains for *189Y* and *449*, respectively), LNs expressing truncated EcR have processes in the ventral region of the AL and SEZ, which is reminiscent of their innervation patterns in 0 h APF pupal brains (*n* = 23 and 9

brains for *189Y* and *449*, respectively). Failed pruning of *189Y*-processes is more prevalent in the central AL, which is likely due to weak GAL4 expression in these LNs, indicating partial penetrance of the phenotype. Scale bars, 20 μm. **c** 0 h APF and 36 h APF pupal brains from control flies (*n* = 12 and 14 ALs for 0 h and 36 h, respectively) and *670-GAL4* driven *UAS-GAL4* flies (*n* = 12 and 14 ALs for 0 h and 36 h, respectively). Sustained GAL4 expression in larval LNs (arrowheads) did not result in labeling of these neurons in 36 h APF pupal brains. Images for *670-GAL4* control flies were reproduced from Fig. 2a. Scale bars, 20 μm.

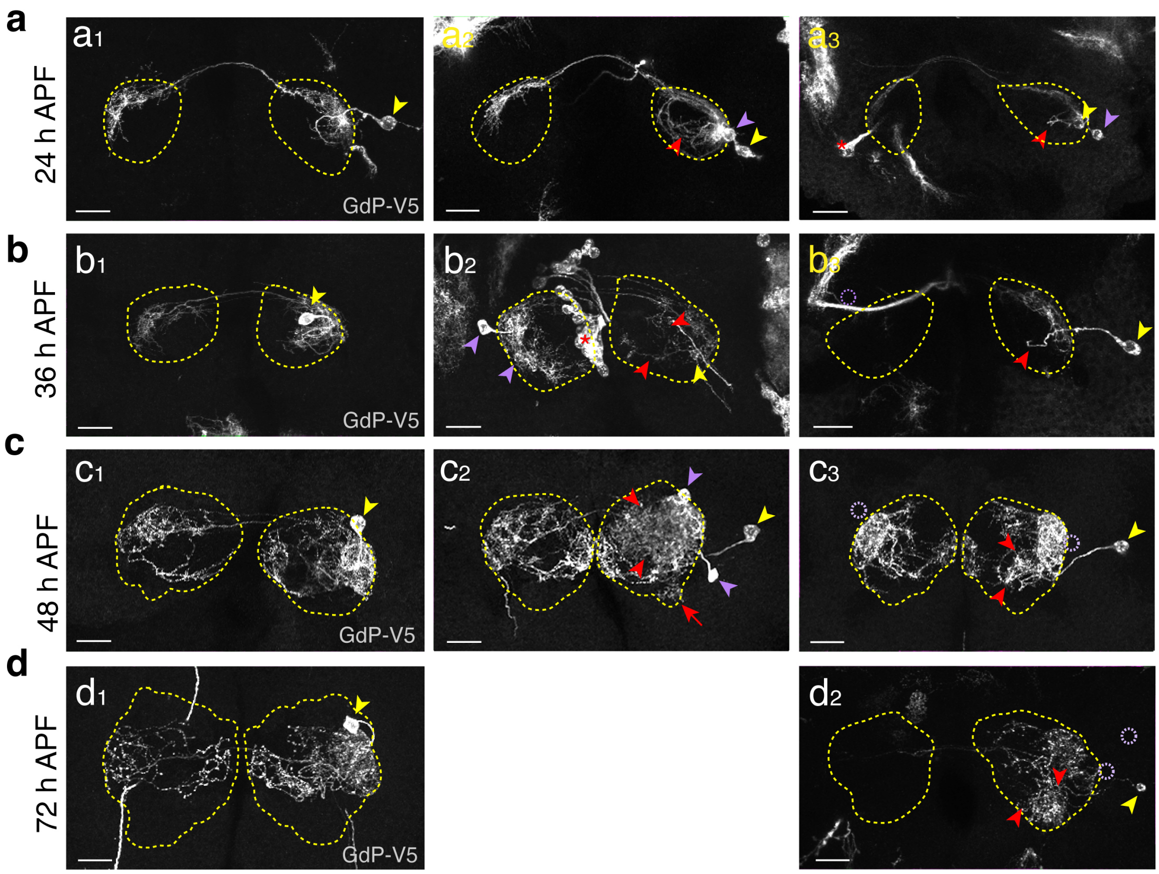

Supplementary Fig. 4 Morphogenesis of *NP3056*-positive larval LNs during pupal development. **a**-**d** The same labeling strategy as in Fig. 4e was used to examine the morphologies of *NP3056*-positive larval LNs in 24 h APF (n = 26 SCs/128 ALs) (**a**), 36 h APF (n = 33 SCs/114 ALs) (**b**), 48 h APF (n = 68 SCs/380 ALs) (**c**) and 72 h APF (n = 53 SCs/245 ALs) (**d**) pupal brains. Brains were stained with V5 and DNcad (not shown) to visualize neurons and neuropil, respectively. These larval LNs may have processes that innervate both ALs (**a**$_1$-**d**$_1$, bilateral LN), the central portion of the AL (**a**$_2$-**c**$_2$) or the posterior of the AL (**a**$_3$-**c**$_3$, **d**$_2$). Middle panels show a presumably regional LN, and right panels show a presumably oligo-glomeruli LN in the right AL, with 1-2 bilateral LNs (magenta arrowheads) in the ipsi- and/or contralateral AL. ALs are indicated by yellow dashed contours. Yellow arrowheads indicate LN soma. Red arrowheads indicate the processes of presumably regional LNs or oligo-glomerular LNs. Red arrow indicates glomerulus V that is likely innervated by the regional LN. Right panels are partial projections of the posterior ALs. Soma that are not covered in the projections are denoted with dashed ovals. Asterisk denotes non-LN cells. Scale bars, 20 µm.

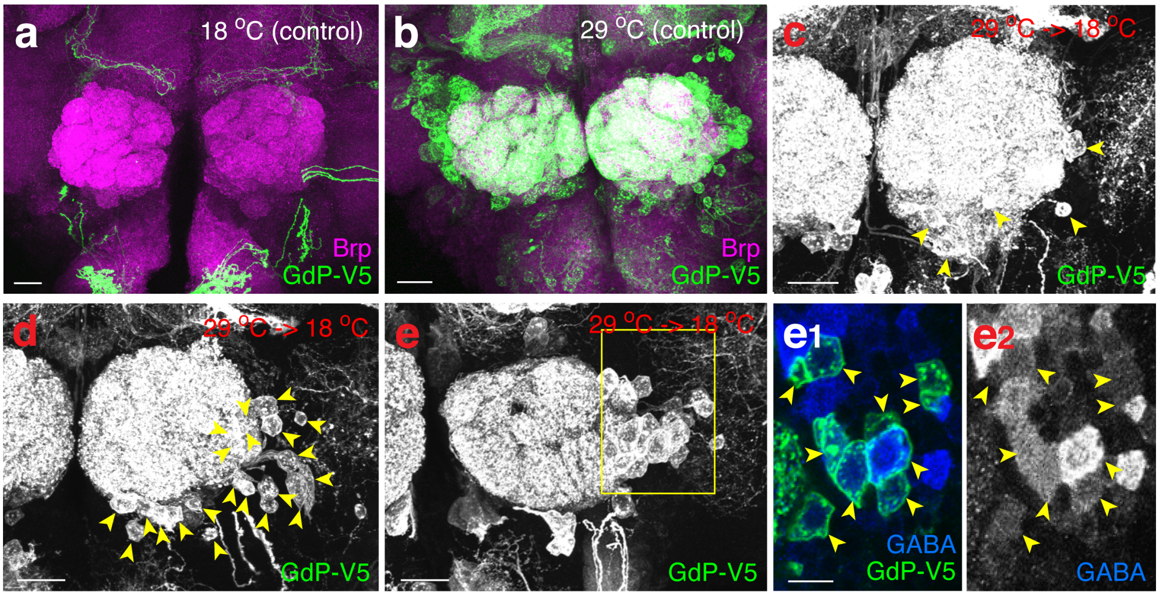

Supplementary Fig. 5 Visualizing *449*-positive larval LNs in adult brains. Similar to Fig. 4a, c but examining *449*-positive larval LNs in adult brains. **a** no GFP-labeled LNs were found in the adult brain of flies raised at a constant 18°C throughout development (n = 35 brains). **b** When the flies were raised at a constant 29°C during development, both larval and adult-specific LNs were labeled (n = 39 brains). **c-e** When flies were raised at 29°C from embryonic stages to larval stage and shifted to 18°C at puparium formation, 3-24 LNs per AL were observed in adult brains (n = 51 brains). **e$_1$**, **e$_2$** A single confocal section of the area outlined in the projected brain shown in **e** reveals that most of labeled 449-positive larval LNs are GABA-positive. Arrowheads indicate the soma of GdP-positive LNs. Scale bars, 20 μm.

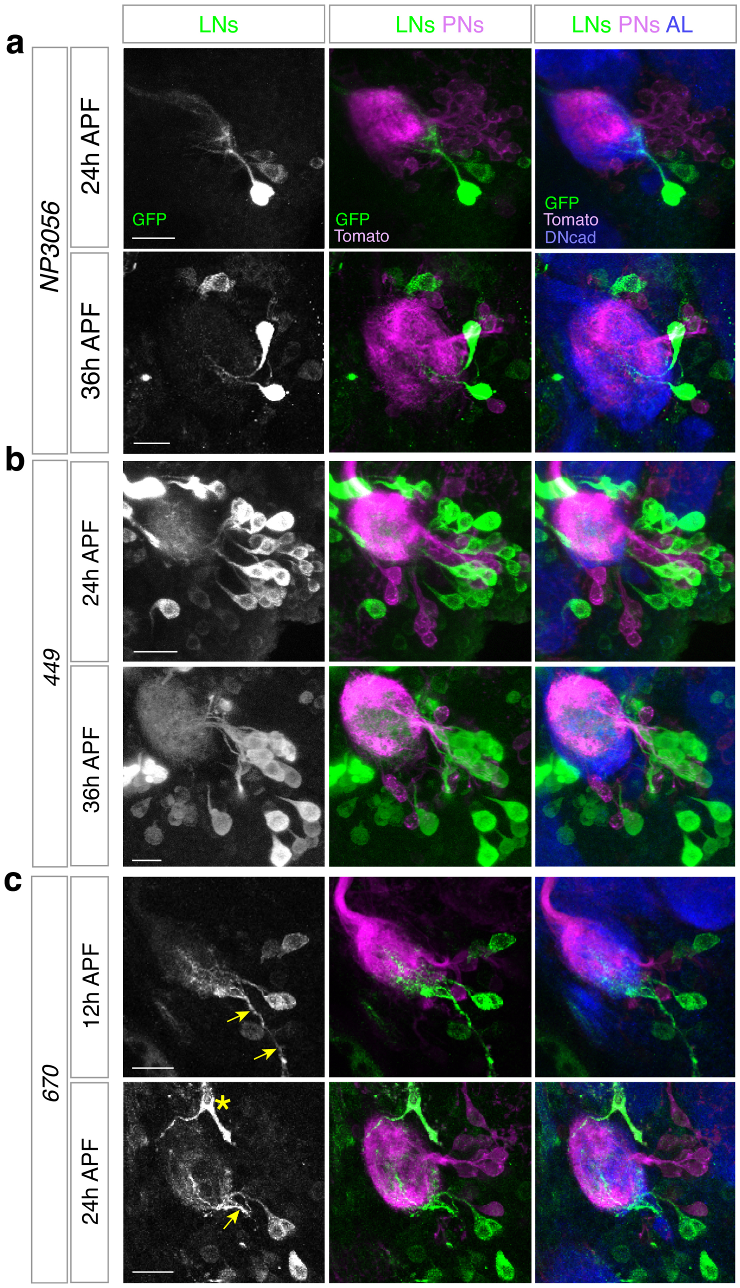

Supplementary Fig. 6 Relative distribution of LN processes and PN dendrites in developing ALs. **a** Partially projected confocal images show pupal brains carrying *NP3056-GAL4* and *GH146-QF*, co-stained with GFP for LNs (white in left, and green in the middle and right panels), mtdT for PNs (magenta in the middle and right panels) and DNcad for neuropils (blue in the right panel) (*n* = 28 and 15 brains at 24 h APF and 36 h APF, respectively). *GH146-QF* is expressed in approximately two-thirds of PNs[6]. **b** Similar to (**a**) but with *449*-positive LNs and *GH146*-positive PNs (*n* = 17 and 20 brains at 24 h APF and 36 h APF, respectively). **c** Similar to (**a**) but with *670*-positive LNs and *GH146*-positive PNs (*n* = 12 and 11 brains at 12 h APF and 24 h APF, respectively). Scale bars, 20 μm.

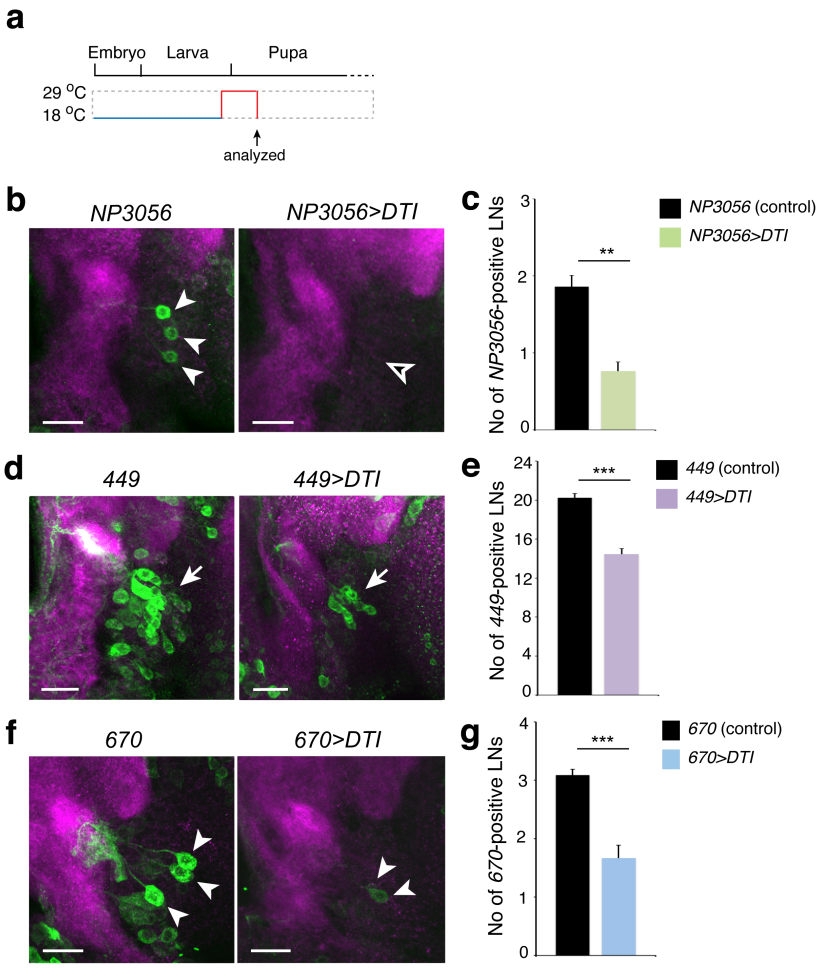

Supplementary Fig. 7 Ectopically expressed DTI effectively ablated larval LNs. **a** Schematic of temperature shift. Larvae were transiently raised at 29°C from late 3rd instar larvae to 6.5 h APF. **b**, **d**, **f** Control or *LN>DTI* pupal brains were stained with DNcad (magenta) and GFP (green). **b** Compared to majority control brains with 2-3 larval LNs (1.86 ± 0.15, *n* = 50 ALs), 75% of *NP3056>DTI* brains have only 0-1 larval LNs (0.76 ± 0.12, *n* = 68 ALs). **d** Compared to control brains with ~20 larval LNs (20.24 ± 0.44, *n* = 34 ALs), *449>DTI* brains have ~14 larval LNs (14.45 ± 0.57, *n* = 29 ALs). **f** Compared to control brains with 3-5 larval LNs (3.09 ± 0.10, *n* = 34 ALs), 70% of *670>DTI* brains have only 0-2 larval LNs (1.67 ± 0.22, *n* = 33 ALs). The majority of larval LNs in the *670>DTI* brains exhibited extremely dim fluorescence and relatively few processes, implying that they were dying. Arrowheads indicate single LNs, arrows indicate a group of LNs, and open arrowhead indicates area that was previously occupied by ablated LNs. Scale bars, 20 μm. **c**, **e**, **g** Quantification of *NP3056*-positive larval LNs (**c**), *449*-positive larval LNs (**e**), and *670*-positive LNs (**g**) in control and *DTI*-expressing brains. The graph shows mean ± s.e.m. Student's *t*-test was used to compare the groups. $^{**}p < 0.005$, $^{***}p < 0.001$.

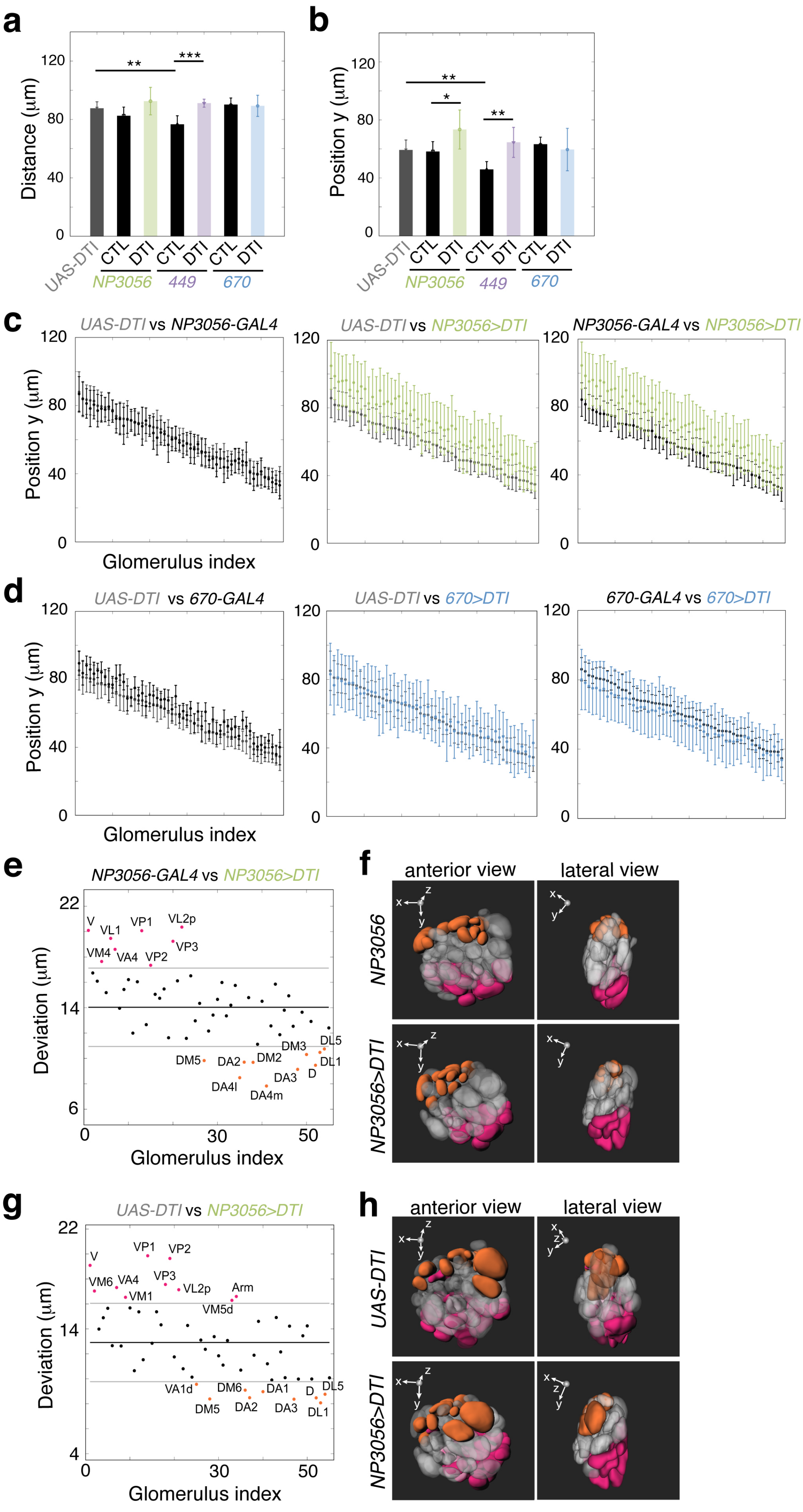
a
Distance (µm)
UAS-DTI
CTL
DTI
NP3056
449
670
b
Position y (µm)
c
UAS-DTI vs NP3056-GAL4
UAS-DTI vs NP3056>DTI
NP3056-GAL4 vs NP3056>DTI
Glomerulus index
d
UAS-DTI vs 670-GAL4
UAS-DTI vs 670>DTI
670-GAL4 vs 670>DTI
e
NP3056-GAL4 vs NP3056>DTI
Deviation (µm)
V
VL1
VP1
VL2p
VP3
VM4
VA4
VP2
DM5
DA2
DM2
DM3
DL5
DL1
DA4l
DA4m
DA3
D
f
anterior view
lateral view
NP3056
NP3056>DTI
g
UAS-DTI vs NP3056>DTI
VM6
VM1
Arm
VM5d
VA1d
DM6
DA1
h
UAS-DTI

Supplementary Fig. 8 Genetic ablation of larval LNs causes aberrant glomeruli organization. **a**, **b** Bar graphs showing the *distance* (**a**) and *y-position* (**b**) of *UAS-DTI* control, *LN-GAL4* control or *LN>DTI* ALs to the reference origin. **c** The y-position of individual glomeruli in *UAS-DTI, NP3056* control and *NP3056>DTI* ALs was determined. Glomeruli were aligned based on their *y-position* value in *UAS-DTI* (left and middle panels) or *UAS-GAL4* (right panel) control ALs. Note that *NP3056>DTI* glomeruli have larger y-position values. The right panel is reproduced from Fig. 6j. **d** Same as (**c**) but for *670>DTI*. *670>DTI* glomeruli do not show obvious differences with *UAS-DTI* or *670* control glomeruli. The right panel is reproduced from Fig. 6k. **e** The relative shift of individual glomeruli in *NP3056>DTI* ALs was quantified as the deviation. Glomeruli were aligned as in the left panel of Fig. 6j. The mean deviation of all glomeruli is 14.0318 (black line). Grey lines indicate one standard deviation (SD) from the mean, which contains 68.3% of the glomeruli. **f** 3D-reconstructed ALs show glomeruli with deviation > 1 SD (pink) and < -1 SD (orange). Identical results in (**e**) and the anterior view of *NP3056* control AL (**f**) were also shown in Fig. 6j. **g**, **h** Same as (**e**) and (**f**), respectively, but *UAS-DTI* was used as control for comparison. The directions of relative X, Y, Z axes are indicated. The axis origin was the tip of the MB peduncle. The information of glomerular index is provided in Supplementary Note 4. **a**-**d**, mean ± s.e.m.

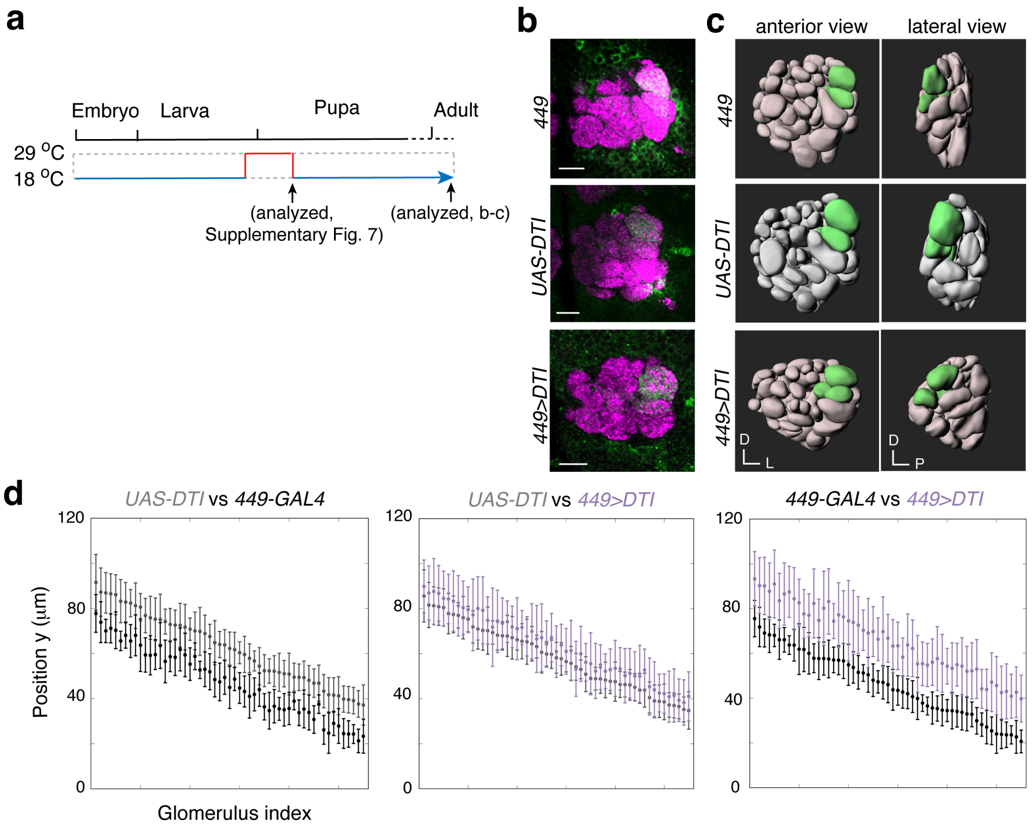

Supplementary Fig. 9 The effect of genetic ablation of *449*-positive larval LNs at a late larval stage on glomeruli organization. **a** Schematic of temperature shift experiments. Larvae were transiently raised at 29°C from late 3rd instar larvae to 6.5 h APF. **b** PN dendrites targeting to glomeruli DA1, VA1d and DC3 of *GAL4* and *UAS-DTI* controls and *449>DTI* brains. Adult brains were stained for Bruchpilot (magenta) to visualize individual glomeruli and GFP to visualize PN dendrites (green). Scale bars, 20 μm. *UAS-DTI* result was reproduced from Fig. 6b. **c** 3D reconstruction of ALs from *449-GAL4* and *UAS-DTI* control (top) and *449>DTI* brains were shown in anterior view (left) and lateral view (right), respectively. D, dorsal; L, lateral; P, posterior. **d** The y-position of individual glomeruli in *449-GAL4* and *UAS-DTI* control (left), *UAS-DTI* control and *449>DTI* (middle) and *449-GAL4* control and *449>DTI* (right) ALs. Glomeruli were aligned based on their y-position value in the *UAS-DTI* (left and middle panels) or *UAS-GAL4* (right panel) control ALs. Note that *449>DTI* glomeruli have similar y-position values to *UAS-DTI* control glomeruli. These results suggest *449-GAL4* alone has some effect on glomerular organization; in other words, it is not clear whether *449>DTI* causing glomerular organization defect or not. The bar graphs show mean ± s.e.m. The information of glomerular index is provided in Supplementary Note 4.

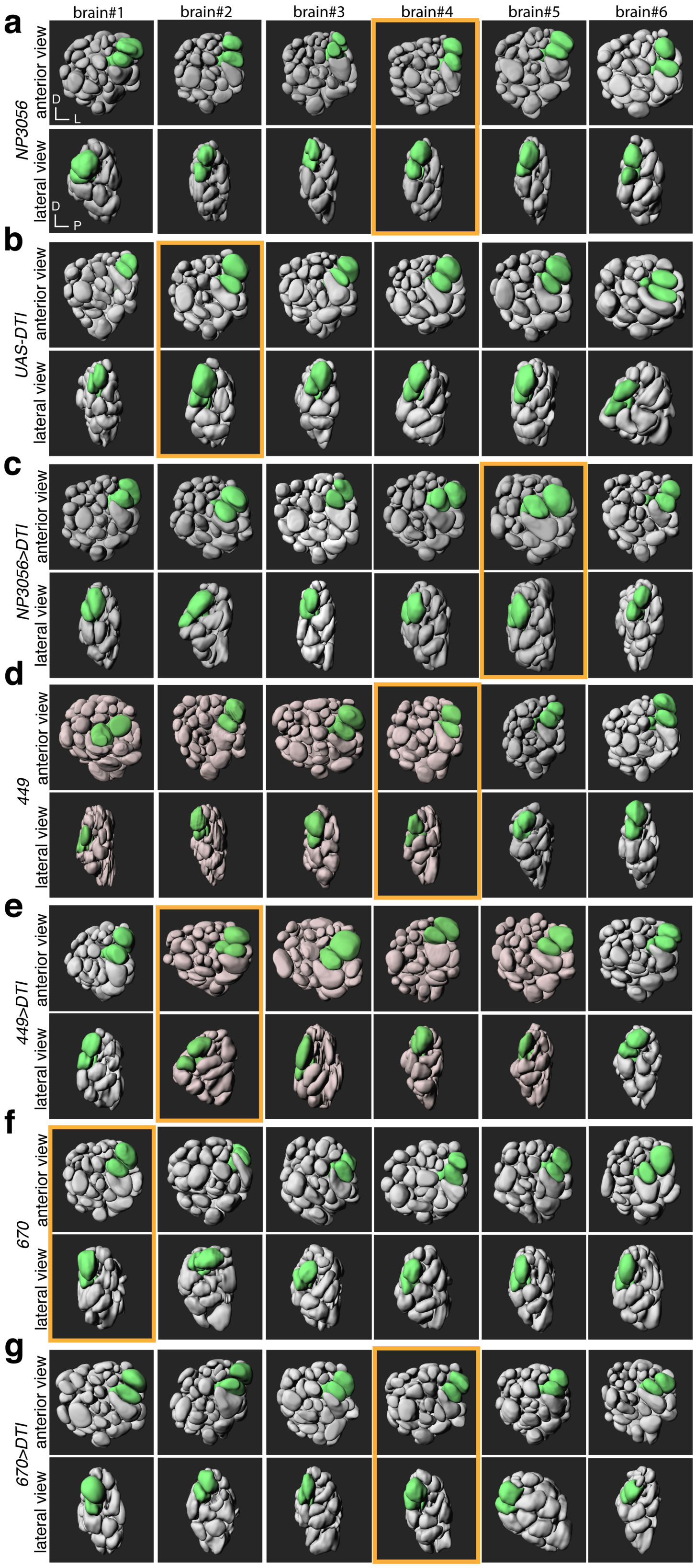

Supplementary Fig. 10 3D reconstruction of ALs from control and *LN>DTI* brains. All 6 reconstructed ALs from control and *LN>DTI* brains were shown in anterior view (top) and lateral view (bottom). The outlined AL of each group was reproduced from Fig. 6c, e and Supplementary Fig. 9c, respectively.

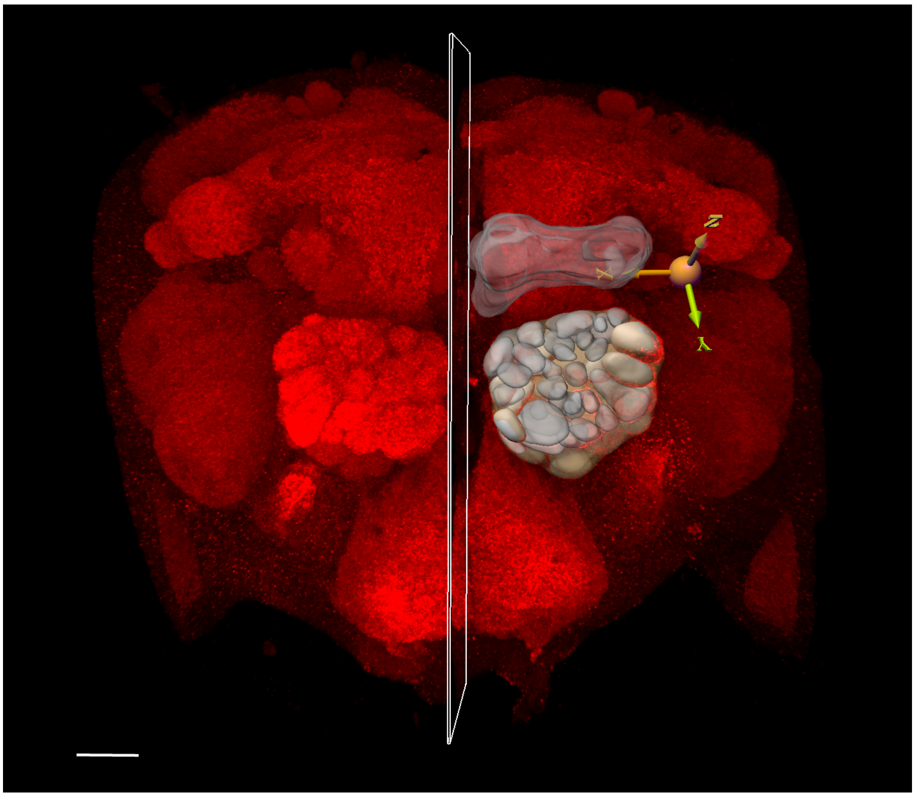

Supplementary Fig. 11 Schematic diagram of a brain 3D reconstruction by Imaris. The surface of individual glomeruli, AL and MB medial lobe were manually outlined (grey) based on neuropil marker (red). The origin for the axes was set at the tip of MB peduncle (orange ball). The Y-Z plane was set such that the two brain hemispheres were evenly divided, with Y-axis parallel to the MB dorsal lobe. Accordingly, Z-axis was perpendicular to the MB dorsal lobe. The X-axis was set perpendicular to the Y-Z plane. Scale bar, 30 μm.

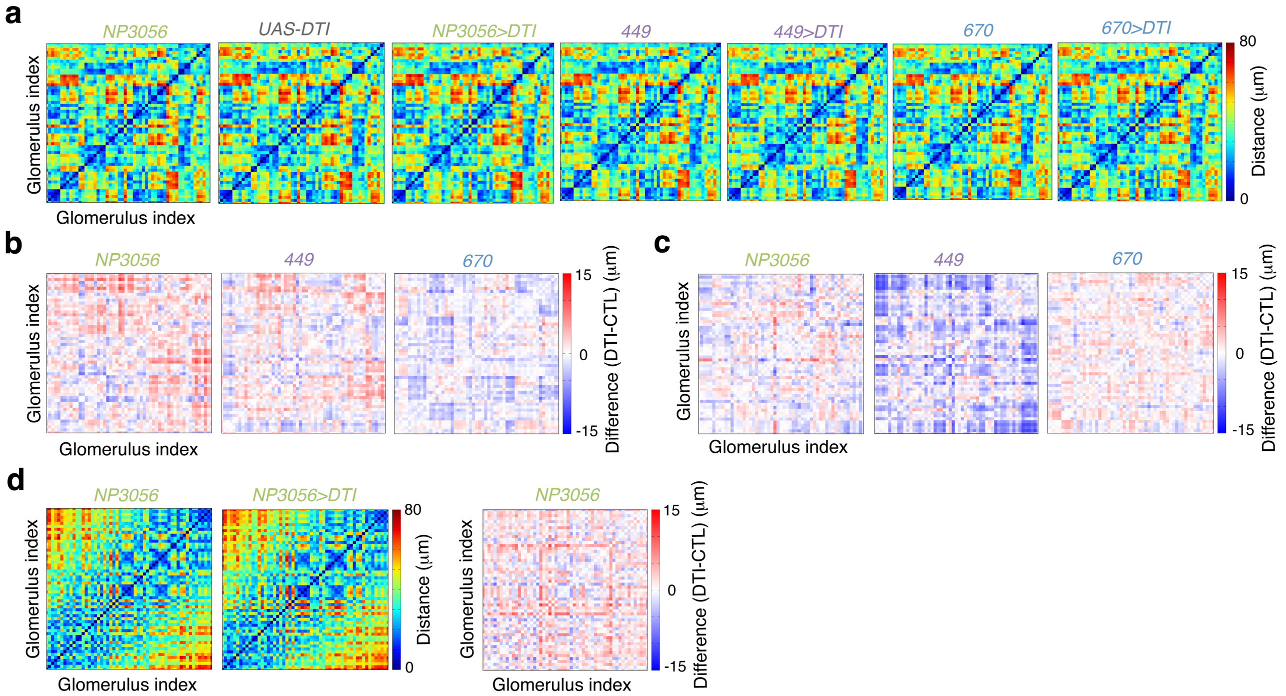

Supplementary Fig. 12 The relative distance between any two glomeruli in the ALs of flies with genetically ablated larval LNs. **a** The relative distance between any two glomeruli in *GAL4* and *UAS-DTI* controls and *LN>DTI* ALs was estimated as in Fig. 6h. Results for *NP3056-GAL4* and *NP3056>DTI* were reproduced from Fig. 6h. **b** The differences between a given pair of glomeruli in *GAL4* control and *LN>DTI* ALs were shown. Results of *NP3056* and *670* were reproduced from Fig. 6h, i. **c** The differences between a given pair of glomeruli in *UAS-DTI* control and *LN>DTI* ALs were shown. **d** The relative distance between any two glomeruli in *NP3056-GAL4* controls and *NP3056>DTI* ALs was estimated and realigned by the glomerulus index used in the y-position shift analysis (Fig. 6j). The result demonstrates no clear correlation between changes of glomerulus-glomerulus distance and y-position shifts. The information of glomerular index is provided in Supplementary Note 4.

Supplementary Table 1 Number of LNs labeled by individual GAL4 lines and their neurotransmitters.

	No of labeled LNs (mean ± s.e.m.)		Neurotransmitter **(Average number of cells)** (mean ± s.e.m.)		
**GAL4**	LNs	AL	GABA+	GABA-	AL
68	2.75 ± 0.16	8	0 ± 0	2.75 ± 0.13	12
95	2.69 ± 0.15	16	0 ± 0	1.8 ± 0.2	5
154	9.43 ± 0.43	7	N.D.*	N.D.	N.D.
191	58.25 ± 1.18	8	N.D.	N.D.	N.D.
330	8.71 ± 0.29	7	N.D.	N.D.	N.D.
351	3.0 ± 0	6	0 ± 0	2.89 ± 0.11	9
361	44.83 ± 5.45	6	N.D.	N.D.	N.D.
382	36.6 ± 2.01	5	18.0 ± 3.03	19.5 ± 2.78	4
388	4.83 ± 0.40	6	N.D.	N.D.	N.D.
415	9.0 ± 0.38	7	N.D.	N.D.	N.D.
421	21.375 ± 0.73	8	9.38 ± 0.91	12.0 ± 0.73	8
449	60.5 ± 1.45	8	53.25 ± 3.89	9.38 ± 4.35	8
501	20.63 ± 0.84	8	18.83 ± 1.85	4.0 ± 0.63	6
551	4.17 ± 0.31	6	N.D.	N.D.	N.D.
584	1.0 ± 0	5	0 ± 0	1.0 ± 0	11
658	30.6 ± 0.95	10	24.63 ± 1.49	2.75 ± 0.88	8
666	19.83 ± 1.30	6	N.D.	N.D.	N.D.
670	3.90 ± 0.18	10	3.0 ± 0.37	1.33 ± 0.42	6
674	27.17 ± 1.17	6	20.11 ± 1.55	6.67 ± 1.04	9
829	23.17 ± 1.35	6	23 ± 1.73	2.67 ± 1.76	3
930	2.73 ± 0.14	11	0 ± 0	2.92 ± 0.08	12
988	23.75 ± 1.25	4	19.67 ± 1.56	3.83 ± 0.40	6
1008	5.33 ± 0.21	6	1.7 ± 0.3	3.4 ± 0.22	10
1078	25.67 ± 0.92	6	12.4 ± 2.87	12.0 ± 1.92	5
1081	94.17 ± 0.21	6	41.5 ± 5.31	29.5 ± 5.30	4

* N.D., not determined.

Supplementary Table 2 Summary of PN dendrite targeting in brains with genetically ablated larval LNs.

Genotype	Reporter of PNs	Glomerular target(s)	Frequency of phenotype (mistargeted glomerulus)	No. of analyzed ALs
UAS-DTI	*MZ19-mCD8GFP*	DA1, VA1d, DC3	10.6 % (1)<br>2.1 % (VA1v)	47
NP3056	*MZ19-mCD8GFP*	DA1, VA1d, DC3	0 %	38
NP3056>DTI	*MZ19-mCD8GFP*	DA1, VA1d, DC3	10 % (VA2)<br>6.7 % (1)<br>3.3 % (VM5v)	30
449	*MZ19-mCD8GFP*	DA1, VA1d, DC3	0%	60
449>DTI	*MZ19-mCD8GFP*	DA1, VA1d, DC3	0%	46
670	*MZ19-mCD8GFP*	DA1, VA1d, DC3	0 %	26
670>DTI	*MZ19-mCD8GFP*	DA1, VA1d, DC3	0%	24

Supplementary Table 3 Statistical test results for Fig. 3h and Supplementary Fig. 7c, 7e and 7g.

Figure	Pair	LN No. (mean ± s.e.m.).	No. of AL	p-value
**Fig. 3h**	*670*	0.03 ± 0.03	36	1.08E-07***
	670>p35	1.95 ± 0.25	20	
**Supplementary Fig. 7c**	*NP3056*	1.86 ± 0.15	50	0.00287***
	NP3056>DTI	0.76 ± 0.12	68	
**Supplementary Fig. 7e**	*449*	20.24 ± 0.44	34	4.33E-11***
	449>DTI	14.45 ± 0.57	29	
**Supplementary Fig. 7g**	*670*	3.09 ± 0.10	34	2.39E-07***
	670>DTI	1.67 ± 0.0.22	33	

Student *t*-test was used. *$p < 0.05$, **$p < 0.01$, ***$p < 0.001$.

Supplementary Table 4 Statistical test results for Fig. 6f and Supplementary Fig. 8a, 8b.

Figure	Pair (No. of AL)	Feature	p-value
GAL4 control vs *GAL4>DTI*			
	NP3056 vs *NP3056>DTI*	Area	0.65
**Supplementary Fig. 8a**	(6 vs 6)	Distance from Origin Reference Frame	0.05578
		Ellipticity (oblate)	0.4088
		Ellipticity (prolate)	0.3885
		Intensity Mean (nc82)	0.812
		Position X Reference Frame	0.7491
**Supplementary Fig. 8b**		Position Y Reference Frame	0.04123*
		Position Z Reference Frame	0.1207
		Sphericity	0.266
**Fig. 6f**		Volume	0.4479
	449 vs *449>DTI*	Area	0.7817
**Supplementary Fig. 8a**	(6 vs 6)	Distance from Origin Reference Frame	0.0008963***
		Ellipticity (oblate)	0.1473
		Ellipticity (prolate)	0.4135
		Intensity Mean (nc82)	0.7231
		Position X Reference Frame	0.7565
**Supplementary Fig. 8b**		Position Y Reference Frame	0.004953**
		Position Z Reference Frame	0.9847
		Sphericity	0.03125*
**Fig. 6f**		Volume	0.7988
	670 vs *670>DTI*	Area	0.8683
**Supplementary Fig. 8a**	(6 vs 6)	Distance from Origin Reference Frame	0.8068
		Ellipticity (oblate)	0.2814
		Ellipticity (prolate)	0.6089
		Intensity Mean (nc82)	0.799
		Position X Reference Frame	0.3887

**Supplementary Fig. 8b**		Position Y Reference Frame	0.5781
		Position Z Reference Frame	0.3289
		Sphericity	0.4506
**Fig 6f**		Volume	0.5079
UAS-DTI control vs *GAL4>DTI*			
	UAS-DTI vs *NP3056>DTI*	Area	0.06171
**Supplementary Fig. 8a**	(6 vs 6)	Distance from Origin Reference Frame	0.2865
		Ellipticity (oblate)	0.5536
		Ellipticity (prolate)	0.7616
		Intensity Mean (nc82)	0.3239
		Position X Reference Frame	0.08424
**Supplementary Fig. 8b**		Position Y Reference Frame	0.05393
		Position Z Reference Frame	0.8542
		Sphericity	0.8318
**Fig. 6f**		Volume	0.02188*
	UAS-DTI vs *449>DTI*	Area	0.3395
**Supplementary Fig. 8a**	(6 vs 6)	Distance from Origin Reference Frame	0.1398
		Ellipticity (oblate)	0.7339
		Ellipticity (prolate)	0.3812
		Intensity Mean (nc82)	0.3269
		Position X Reference Frame	0.8169
**Supplementary Fig. 8b**		Position Y Reference Frame	0.3284
		Position Z Reference Frame	0.9211
		Sphericity	0.8098
**Fig. 6f**		Volume	0.2795
	UAS-DTI vs *670>DTI*	Area	0.3781
**Supplementary Fig. 8a**	(6 vs 6)	Distance from Origin Reference Frame	0.6474
		Ellipticity (oblate)	0.7125
		Ellipticity (prolate)	0.884

		Intensity Mean (nc82)	0.2201
		Position X Reference Frame	0.9664
**Supplementary Fig. 8b**		Position Y Reference Frame	0.966
		Position Z Reference Frame	0.5201
		Sphericity	0.1854
**Fig. 6f**		Volume	0.02084*
UAS-DTI control vs *GAL4* control			
	UAS-DTI vs *NP3056*	Area	0.04082*
**Supplementary Fig. 8a**	(6 vs 6)	Distance from Origin Reference Frame	0.1208
		Ellipticity (oblate)	0.2735
		Ellipticity (prolate)	0.541
		Intensity Mean (nc82)	0.2964
		Position X Reference Frame	0.08935
**Supplementary Fig. 8b**		Position Y Reference Frame	0.7893
		Position Z Reference Frame	0.3107
		Sphericity	0.1504
**Fig. 6f**		Volume	0.1043
	UAS-DTI vs *449*	Area	0.8559
**Supplementary Fig. 8a**	(6 vs 6)	Distance from Origin Reference Frame	0.004897**
		Ellipticity (oblate)	0.297
		Ellipticity (prolate)	0.9678
		Intensity Mean (nc82)	0.3858
		Position X Reference Frame	0.4899
**Supplementary Fig. 8b**		Position Y Reference Frame	0.004188**
		Position Z Reference Frame	0.9282
		Sphericity	0.02187*
**Fig. 6f**		Volume	0.4226
	UAS-DTI vs *670*	Area	0.5616
**Supplementary Fig. 8a**	(6 vs 6)	Distance from Origin Reference Frame	0.3505

	Ellipticity (oblate)	0.7273
	Ellipticity (prolate)	0.543
	Intensity Mean (nc82)	0.1719
	Position X Reference Frame	0.367
**Supplementary Fig. 8b**	Position Y Reference Frame	0.2779
	Position Z Reference Frame	0.1733
	Sphericity	0.1232
**Figure 6f**	Volume	0.7897

Student *t*-test was used. *$p < 0.05$, **$p < 0.01$, ***$p < 0.001$.

Supplementary Table 5 Genotypes of flies used in experiments described in Fig. 1-6 and Supplementary Fig. 1-8.

Figure	Genotype
**Fig. 1b**	(330-GAL4, 388-GAL4)<br>*w, IS-GAL4/y w; UAS-nuclacZ UAS-mCD8GFP/+; ;*
	(95-GAL4, 154-GAL4, 351-GAL4, 1078-GAL4, 1081-GAL4)<br>*y w/w; IS-GAL4/UAS-nuclacZ UAS-mCD8GFP; ;*
	(68-GAL4, 191-GAL4, 361-GAL4, 382-GAL4, 415-GAL4, 421-GAL4,449-GAL4, 501-GAL4,551-GAL4, 584-GAL4, 658-GAL4, 666-GAL4, 670-GAL4, 674-GAL4, 829-GAL4, 930-GAL4, 988-GAL4, 1008-GAL4)<br>*y w/w; UAS-nuclacZ UAS-mCD8GFP/+; IS-GAL4/+;*
**Fig. 2a**	*(y) w; 189Y-GAL4/UAS-nuclacZ UAS-mCD8GFP;+;*
	(y) w; UAS-nuclacZ UAS-mCD8GFP/+; NP3056/+;
	y w/w; UAS-nuclacZ UAS-mCD8GFP/+; 449-GAL4/+;
	y w/w; UAS-nuclacZ UAS-mCD8GFP/+; 670-GAL4/+;
**Fig. 2b**	*y w/w; UAS-nuclacZ UAS-mCD8GFP/+; 501-GAL4/+;*
	y w/w; 351-GAL4/UAS-nuclacZ UAS-mCD8GFP; ;
**Fig. 2c**	*y w/w; UAS-nuclacZ UAS-mCD8GFP/+; 988-GAL4/+;*
	y w/w; 95-GAL4/UAS-nuclacZ UAS-mCD8GFP; ;
**Fig. 3a**	*w; UAS-GAL4 (12B)/+; UAS-Kaeda.A (3)/NP3056;*
**Fig. 3c**	*(y) w; UAS-FLP/13xLexAop2(FRT. stop)myr::smGdP-V5 (attp40); NP3056/tubP-GAL80[ts] (2), nSyb-LexA.DBD::QF.AD (attP2)*
**Fig. 3d**	(control)<br>*(y) w; tubP-GAL80[ts] (20)/UAS-mCD8GFP.1; NP3056/+;*
	(EcRDN)<br>*(y) w; UAS-EcR.B1-ΔC655,F645A (TP1), tubP-GAL80[ts] (20)/UAS-mCD8GFP.1; NP3056/+;*
**Fig. 3e**	*(y) w; UAS-FLP/QUAS(FRT.stop)mCD8GFP.P (10); 670-GAL4/tubP-GAL80[ts] (7), nSyb-QF2w.P (attP2);*
**Fig. 3f**	*y w/w; UAS-mCD8GFP/+; 670-GAL4/+;*
**Fig. 3g**	(control)<br>*ey3.5-GAL80, w/w; tubP-GAL80[ts] (20)/UAS-mCD8GFP; 670-GAL4/+;*
	(p35)<br>*ey3.5-GAL80, w/w; tubP-GAL80[ts] (20)/UAS-mCD8GFP.1/+; UAS-p35.H (BH2)/670-GAL4;*

**Fig. 4b**	*w[1118] hs-FLPG5.PEST (attP3)/w; 189Y-GAL4/+; 10xUAS(FRT.stop)myr::smGdP-HA (VK00005),10xUAS-(FRT.stop)myr::smGdP-V5-THS-10xUAS(FRT.stop)myr::smGdP-FLAG (su(Hw)attp1)/+;*
**Fig. 4c**	*(y) w; UAS-Flp/189Y-GAL4; 13xLexAop2(FRT.stop)myr::smGdP-HA (VK00005)/tubP-GAL80[ts] (2), nSyb-LexA.DBD::QF.AD (attP2);*
**Fig. 4d**	*w[1118] hs-FLPG5.PEST (attP3)/w;; NP3056-GAL4/ 10xUAS(FRT.stop)myr::smGdP-HA (VK00005),10xUAS-(FRT.stop)myr::smGdP-V5-THS-10xUAS(FRT.stop)myr::smGdP-FLAG (su(Hw)attp1);*
**Fig. 4e**	*(y) w; UAS-FLP/13xLexAop2(FRT. stop)myr::smGdP-V5 (attp40); NP3056/tubP-GAL80[ts] (2), nSyb-LexA.DBD::QF.AD (attP2);*
**Fig. 5a-d**	*(y) w; UAS-Flp/189Y-GAL4; 13xLexAop2(FRT.stop)myr::smGdP-HA (VK00005)/tubP-GAL80[ts] , nSyb-LexA.DBD::QF.AD (attP2);*
**Fig. 6b, f**	(*GAL4* control)<br>*ey3.5-GAL80, w/y w; tubP-GAL80[ts] (20)/MZ19-mCD8GFP[y+]; NP3056-GAL4/+;*
	(*UAS-DTI* control)<br>*ey3.5-GAL80, w/y w; UAS-DTI (18), tubP-GAL80[ts] (20)/MZ19-mCD8GFP[y+];;*
	(*DTI*)<br>*ey3.5-GAL80, w/y w; UAS-DTI (18), tubP-GAL80[ts] (20)/MZ19-mCD8GFP[y+]; NP3056-GAL4/+;*
**Fig. 6d, f**	(control)<br>*ey3.5-GAL80/ey3.5-GAL80; tubP-GAL80[ts] (20)/MZ19-mCD8GFP(y+); 670-GAL4/+*
	(*DTI*)<br>*ey3.5-GAL80/+; UAS-DTI (18), tubP-GAL80[ts] (20)/MZ19-mCD8GFP(y+); 670-GAL4/+;*
**Fig. 6f**	(control)<br>*ey3.5-GAL80, w/y w; tubP-GAL80[ts] (20)/MZ19-mCD8GFP[y+]; 449-GAL4/+;*
	(*DTI*)<br>*ey3.5-GAL80, w/y w; UAS-DTI (18), tubP-GAL80[ts] (20)/MZ19-mCD8GFP[y+]; 449-GAL4/+;*
**Supplementary Fig. 1a**	*(y) w; UAS-nuclacZ UAS-mCD8GFP/+; krasavietz-GAL4/+;*
	LCCH3-GAL4/y w; UAS-nuclacZ UAS-mCD8GFP; ;
	(y) w; 189Y-GAL4/UAS-nuclacZ UAS-mCD8GFP; ;
	(y) w; UAS-nuclacZ UAS-mCD8GFP/+; NP3056-GAL4/+;
	y w/w; 1081-GAL4/UAS-nuclacZ UAS-mCD8GFP; ;
	(*421-GAL4, 449-GAL4, 658-GAL4, 670-GAL4*)<br>*y w/+; UAS-nuclacZ UAS-mCD8GFP/+; IS-GAL4/+;*

**Supplementary Fig. 1b**	(351-GAL4, 1078-GAL4)<br>*y w/w; IS-GAL4/UAS-nuclacZ UAS-mCD8GFP; ;*
	(382-GAL4, 501-GAL4, 674-GAL4, 1008-GAL4)<br>*y w/w; UAS-nuclacZ UAS-mCD8GFP/+; IS-GAL4/+;*
**Supplementary Fig. 1c**	*y w/w; 95-GAL4/UAS-nuclacZ UAS-mCD8GFP; ;*
	(68-GAL4, 930-GAL4, 988-GAL4)<br>*y w/w; UAS-nuclacZ UAS-mCD8GFP/+; IS-GAL4/+;*
**Supplementary Fig. 2a**	*(y) w; 189Y-GAL4/UAS-nuclacZ UAS-mCD8GFP; ;*
	(y) w; UAS-nuclacZ UAS-mCD8GFP/+; NP3056-GAL4/+;
	(y) w; UAS-nuclacZ UAS-mCD8GFP/+; 449-GAL4/+;
	(y) w; UAS-nuclacZ UAS-mCD8GFP/+; 670-GAL4/+;
	(y) w; c739-GAL4/UAS-nuclacZ UAS-mCD8GFP;;
	(y) w; 189Y-GAL4/UAS-nuclacZ UAS-mCD8GFP; NP3056-GAL4/+;
	(y) w; 189Y-GAL4/UAS-nuclacZ UAS-mCD8GFP; 449-GAL4/+;
	(y) w; 189Y-GAL4/UAS-nuclacZ UAS-mCD8GFP; 670-GAL4/+;
	(y) w; 189Y-GAL4/c739-GAL4 UAS-nuclacZ UAS-mCD8GFP;;
	(y) w; UAS-nuclacZ UAS-mCD8GFP/+; NP3056-GAL4 449-GAL4/+
	(y) w; 189Y-GAL4/UAS-nuclacZ UAS-mCD8GFP; NP3056-GAL4 449-GAL4/+
	(y) w; UAS-nuclacZ UAS-mCD8GFP/+; NP3056-GAL4, 449-GAL4/670-GAL4;
	(y) w; 189Y-GAL4/UAS-nuclacZ UASmCD8GFP; NP3056-GAL4, 449-GAL4/670-GAL4;
	(y) w; c739-GAL4/UAS-nuclacZ UAS-mCD8GFP; NP3056-GAL4, 449-GAL4/670-GAL4;
**Supplementary Fig. 3b**	*(y) w; 189Y-GAL4/UAS-nuclacZ UAS-mCD8GFP; ;*
	y) w; UAS-nuclacZ UAS-mCD8GFP/+; NP3056/+;
	(y) w; UAS-nuclacZ UAS-mCD8GFP/+;449-GAL4/+;
**Supplementary Fig. 3b**	(*189Y*, control)<br>*(y) w; 189Y-GAL4 UAS-mCD8GFP/CyO; DsRed; +* (or)<br>*(y) w; UAS-nuclacZ, UAS-mCD8GFP/189Y-GAL4; +*
	(*189Y, EcRDN*)<br>*(y) w; UAS-EcR.B1-ΔC655,F645A (TP1)/189Y-GAL4, UAS-mCD8GFP.1; ;*
	(*449*, control)<br>*(y) w; UAS-mCD8GFP.1/CyO, DsRed; 449-GAL4/+* (or)<br>*(y) w; UAS-mCD8GFP.1/+; 449-GAL4/+;*

	(*449, EcRDN*)<br>*(y) w; UAS-EcR.B1-ΔC655,F645A (TP1)/ UAS-mCD8GFP.1; 449-GAL4/+;*
**Supplementary Fig. 3c**	(control)<br>*y w/w; UAS-nuclacZ UAS-mCD8GFP/+; 670-GAL4/+*<br>(*UAS-GAL4*)<br>*w; UAS-GAL4.H (12B) UAS-mCD8GFP/+; 670-GAL4/+;*
**Supplementary Fig. 4a-d**	*(y) w; UAS-FLP/13xLexAop2(FRT. stop)myr::smGdP-V5 (attp40); NP3056/tubP-GAL80[ts] (2), nSyb-LexA.DBD::QF.AD (attP2);*
**Supplementary Fig. 5a-e**	*(y) w; UAS-FLP/13xLexAop2(FRT. stop)myr::smGdP-V5 (attp40); 449/tubP-GAL80[ts] (2), nSyb-LexA.DBD::QF.AD (attP2);*
**Supplementary Fig. 6a**	*(y) w; UAS-mCD8GFP/+; NP3056-GAL4/GH146-QF (53), QUAS-mtdTomato-3xHA (24);*
**Supplementary Fig. 6b**	*(y) w; UAS-mCD8GFP/+; 449-GAL4/GH146-QF (53), QUAS-mtdTomato-3xHA (24);*
**Supplementary Fig. 6c**	*(y) w; UAS-mCD8GFP/+; 670-GAL4/GH146-QF (53), QUAS-mtdTomato-3xHA (24);*
**Supplementary Fig. 7b**	(control)<br>*ey3.5-GAL80, w/y w; tubP-GAL80[ts] (20)/UAS-mCD8GFP.1; NP3056/+;*
	(*DTI*)<br>*ey3.5-GAL80, w/y w; UAS-DTI (18), tubP-GAL80[ts] (20)/UAS-mCD8GFP.1; NP3056/+;*
**Supplementary Fig. 7d**	(control)<br>*ey3.5-GAL80, w/y w; tubP-GAL80[ts] (20)/UAS-mCD8GFP.1; 449-GAL4/+;*
	(*DTI*)<br>*ey3.5-GAL80, w/y w; UAS-DTI (18), tubP-GAL80[ts] (20)/UAS-mCD8GFP.1; 449-GAL4/+;*
**Supplementary Fig. 7f**	control)<br>*ey3.5-GAL80/ey3.5-GAL80; tubP-GAL80[ts] (20)/UAS-mCD8GFP.1; 670-GAL4/+*
	(*DTI*)<br>*ey3.5-GAL80/+; UAS-DTI (18), tubP-GAL80[ts] (20)/UAS-mCD8GFP.1; 670-GAL4/+;*
**Supplementary Fig. 9b**	(*GAL4* control)<br>*ey3.5-GAL80, w/y w; tubP-GAL80[ts] (20)/MZ19-mCD8GFP[y+]; 449-GAL4/+;*
	(*UAS-DTI* control)<br>*ey3.5-GAL80, w/y w; UAS-DTI (18), tubP-GAL80[ts] (20)/MZ19-mCD8GFP[y+];;*
	(*DTI*)<br>*ey3.5-GAL80, w/y w; UAS-DTI (18), tubP-GAL80[ts] (20)/MZ19-mCD8GFP[y+]; 449-GAL4/+;*

Supplementary Table 6 Secondary antibodies used in this study.

Antibody	Catalogue number	Working dilution
Goat anti-mouse IgG (H+L) Alexa Flour® 488	Jackson ImmunoResearch Laboratories (115-545-166)	1:500
Goat anti-mouse IgG (H+L) DyLight™ 488	Jackson ImmunoResearch Laboratories (115-485-166)	1:500
Goat anti-mouse IgG (H+L) Cy™3	Jackson ImmunoResearch Laboratories (115-165-166)	1:500
Goat anti-mouse IgG (H+L) DyLight™ 649	Jackson ImmunoResearch Laboratories (115-495-166)	1:500
Goat anti-mouse IgG (H+L) Alexa Fluor® 674	Jackson ImmunoResearch Laboratories (115-605-166)	1:500
Goat anti-rabbit IgG (H+L) Alexa Flour® 488	Invitrogen (A-11034)	1:500
Goat anti-rabbit IgG (H+L) Alexa Flour® 488	Jackson ImmunoResearch Laboratories (111-545-144)	1:500
Goat anti-rabbit IgG (H+L) DyLight™ 488	Jackson ImmunoResearch Laboratories (111-485-144)	1:500
Goat anti-rabbit IgG (H+L) Cy™3	Jackson ImmunoResearch Laboratories (111-165-144)	1:500
Goat anti-rabbit IgG (H+L) Alexa Fluor® 647	Jackson ImmunoResearch Laboratories (111-605-144)	1:500
Goat anti-rabbit IgG (H+L) DyLight™ 649	Jackson ImmunoResearch Laboratories (111-495-144)	1:500
Goat anti-rat IgG (H+L) DyLight™ 488	Jackson ImmunoResearch Laboratories (112-485-167)	1:500
Goat Anti-rat IgG (H+L) Alexa Fluor® 488	Jackson ImmunoResearch Laboratories (112-545-167)	1:500
Goat anti-rat IgG (H+L) Cy™3	Jackson ImmunoResearch Laboratories (112-165-167)	1:500
Goat Anti-rat IgG (H+L) Alexa Fluor® 647	Jackson ImmunoResearch Laboratories (112-605-167)	1:500
Goat anti-rat IgG (H+L) DyLight™ 649	Jackson ImmunoResearch Laboratories (112-495-167)	1:500

Supplementary References

1 Chou, Y. H. *et al.* Diversity and wiring variability of olfactory local interneurons in the Drosophila antennal lobe. *Nat Neurosci* **13**, 439-449, doi:nn.2489 [pii]10.1038/nn.2489 (2010).

2 Lin, S., Kao, C. F., Yu, H. H., Huang, Y. & Lee, T. Lineage analysis of Drosophila lateral antennal lobe neurons reveals notch-dependent binary temporal fate decisions. *PLoS Biol* **10**, e1001425, doi:10.1371/journal.pbio.1001425 (2012).

3 Lai, S. L., Awasaki, T., Ito, K. & Lee, T. Clonal analysis of Drosophila antennal lobe neurons: diverse neuronal architectures in the lateral neuroblast lineage. *Development* **135**, 2883-2893, doi:dev.024380 [pii]10.1242/dev.024380 (2008).

4 Thum, A. S., Leisibach, B., Gendre, N., Selcho, M. & Stocker, R. F. Diversity, variability, and suboesophageal connectivity of antennal lobe neurons in D. melanogaster larvae. *J Comp Neurol* **519**, 3415-3432, doi:10.1002/cne.22713 (2011).

5 Ramaekers, A. *et al.* Glomerular maps without cellular redundancy at successive levels of the Drosophila larval olfactory circuit. *Curr Biol* **15**, 982-992 (2005).

6 Potter, C. J., Tasic, B., Russler, E. V., Liang, L. & Luo, L. The Q system: a repressible binary system for transgene expression, lineage tracing, and mosaic analysis. *Cell* **141**, 536-548, doi:S0092-8674(10)00178-9 [pii]10.1016/j.cell.2010.02.025 (2010).
